# COVID-19 mRNA Vaccines Are Generally Safe in the Short Term: A Vaccine Vigilance Real-World Study Says

**DOI:** 10.3389/fimmu.2021.669010

**Published:** 2021-05-21

**Authors:** Gang Chen, Xiaolin Li, Meixing Sun, Yangzhong Zhou, Meifang Yin, Bin Zhao, Xuemei Li

**Affiliations:** ^1^ Nephrology Department, Peking Union Medical College Hospital, Peking Union Medical College, Chinese Academy of Medical Sciences, Beijing, China; ^2^ Pharmacy Department, Peking Union Medical College Hospital, Peking Union Medical College, Chinese Academy of Medical Sciences, Beijing, China; ^3^ State Key Laboratory of Bioactive Substance and Function of Natural Medicines, Institute of Materia Medica, Peking Union Medical College, Chinese Academy of Medical Sciences, Beijing, China

**Keywords:** COVID-19, vaccine, mRNA, safety issue, adverse event

## Abstract

**Background:**

The prophylactic vaccination of COVID-19 mRNA vaccines is the first large-scale application of this kind in the human world. Over 1.8 million doses of the COVID-19 vaccine had been administered in the US until December 2020, and around 0.2% submitted AE reports to the Vaccine Adverse Event Reporting System (VAERS). This study aimed to evaluate the AEs following immunization (AEFIs) and analyze the potential associations based on the information from the VAERS database.

**Methods:**

We searched the VAERS database recorded AEFIs after COVID-19 vaccines in December 2020. After data mapping, we summarized demographic and clinical features of reported cases. Fisher exact test was used to comparing the clinical characteristics among AE groups with an anaphylactic response, concerning neurological disorders and death.

**Results:**

VAERS reported 3,908 AEFIs of COVID-19 vaccines in December 2020. Most (79.68%) were reported after the first dose of the vaccine. Among the reported cases, we found that general disorders (48.80%), nervous system disorders (46.39%), and gastrointestinal disorders (25.54%) were the most common AEFIs. The allergy history was more frequent in vaccine recipients with anaphylactic reactions than those without (64.91% *vs.* 49.62%, OR = 1.88, *P <0.017*). History of anxiety or depression was more common in subjects reporting severe neurological AEFIs than those reporting other AEFIs (18.37% *vs.* 7.85%, OR = 2.64, *P <0.017*). Cases reporting death were significantly older (79.36 ± 10.41-year-old *vs.* 42.64 ± 12.55-year-old, *P <0.01*, 95% CI 29.30–44.15) and more likely experienced hypertension (50.00% *vs.* 11.42%, OR = 7.76, *P <0.01*) and neurological disorders (50.00% *vs.* 5.36%, OR = 17.65, *P <0.01*) than other vaccine recipients. The outpatient and emergency room visit rates were 11.92 and 22.42% for AEFIs, and 2.53% of cases needed hospitalization.

**Conclusion:**

AEFIs of COVID-19 mRNA vaccines were generally non-severe local or systemic reactions. A prior allergy history is the risk factor for anaphylaxis, while a history of anxiety may link with severe neurological AEs. Such vaccine recipients need further evaluation and monitor.

## Background

As of the middle of February 2021, around 1.3% of the world’s population has been infected with coronavirus disease 2019 (COVID-19), and over 2.3 million people have died (1). One person has been infected every 7.7 s, on average, since the start of 2021. To combat the pandemic, global efforts to produce COVID-19 vaccines have gained momentum and generated more than 240 vaccine candidates in clinical or preclinical stages, updated to February 2021 (2). The United States (US) Food and Drug Administration (FDA) issued Emergency Use Authorization for Pfizer-BioNTech (BNT162b2) and Moderna (mRNA-1273) COVID-19 vaccines separately on December 11th and 18th, 2020. The head-to-head battle between human beings and COVID-19 has entered a new stage.

Other than traditional whole-pathogen vaccines, Pfizer-BioNTech and Moderna adopted new generation technology and commercialized messenger RNA (mRNA) vaccines. Such vector-based vaccines only incorporate specific antigens from the pathogen, namely the spike protein fragment of the severe acute respiratory syndrome coronavirus 2 (SARS-CoV-2). Theoretically, vector-based vaccines process better safety profiles (3); both BNT162b2 and mRNA-1273 phase 3 randomized controlled trials (RCTs) have demonstrated the low incidence (0.5–0.6%) of serious adverse events (AEs) in mRNA vaccine groups (4, 5). Most of the AEs reported by the mRNA vaccine recipients were self-limited local and systemic reactions, such as injection site pain (71–84%), fatigue (33–51%), and headache (37–39%) (4, 5).

The past months have witnessed the first large-scale application of mRNA vaccines in the human world. Concerns still exist since the occasional reports of severe or concerning AEs like anaphylaxis (6, 7), virus infection in patients with immune diseases (8), facial nerve palsy (9, 10), and even death (9). As a complement to clinical trials, real-world AE monitoring is crucial to expand our understandings of suspected AEs following immunization (AEFIs). Until December 2020, more than 1.8 million doses of the COVID-19 mRNA vaccine had been administered in the US, and around 0.2% of AE reports had been submitted to the Vaccine Adverse Event Reporting System (VAERS) (7); the reported cases were fairly sufficient to conduct certain informative analyses. We aimed to evaluate the AEs following the mRNA vaccines and further analyzed the potential associations between the clinical backgrounds and some certain AEFIs, including anaphylactic response, concerning neurological disorders and death.

## Method

### Database Description and Data Mining

The VAERS database (https://vaers.hhs.gov) is a US vaccine safety surveillance program established in 1990 run by the Centers for Disease Control and Prevention (CDC) and FDA. VAERS serves as an early warning system to detect possible safety issues with US-licensed vaccines by collecting information about AEs (possible side effects or health problems) that occur after vaccination. The vaccine recipients, health care providers, and vaccine manufacturers are welcomed to report AEs to VAERS. By law, vaccine manufacturers are required to report AEs that come to their attention, and healthcare professionals are required to report AEs that are considered a contraindication to further doses of vaccine (11); therefore, the chance of neglecting an important and concerning AEs would be reduced. Although VAERS accepts all reports without rendering judgment on clinical importance or whether the vaccine might have caused the AE, VAERS is of great importance as a hypothesis-generating system with the primary goal to detect safety signals that might be related to vaccination (11).

VAERS data contain the following files: VAERSDATA.CSV which includes reports and patient information, VAERSVAX.CSV which includes vaccine information, and VAERSSYMPTOMS.CSV which includes the AEs. Patient information contains demographic data, current illnesses, past histories and allergy histories. Vaccine information contains vaccine manufacturers and providers. AE information contains vaccination date, symptom onset date, AE description, and prognosis. We defined time to onset of AEs by calculating the interval between vaccination date and symptom onset date. We further coded AE description utilizing the Preferred Terms (PTs) of the Medical Dictionary for Regulatory Activities (MedDRA) dictionary, a clinically validated, internationally standardized terminology.

We included reports received by the VAERS of COVID-19 mRNA Vaccines in December 2020, which were retrieved from the 2020 Zip File of VAERS Data Sets (Date of download: January 15th, 2021).

### Data Mapping

We used the MedDRA Version 23.1 for AEs mapping. The “System Organ Classes” (SOCs) (https://www.meddra.org) were used for AEs classifications. The SOC is an international classification system that is grouped by etiology (e.g., Infections and infestations), manifestation site (e.g., Gastrointestinal disorders), or purpose (e.g., Surgical and medical procedures). The medications in VAERS can be registered arbitrarily; therefore, we utilized MICROMEDEX as a dictionary for the currently administrated medications. All VAERS medication inputs were referred to MICROMEDEX and categorized.

Two physicians with more than seven years of experience independently analyzed the current illnesses, past and allergy histories described with natural language in the VAERS and categorized them by organ system. If their classification results were different, a professor of internal medicine with more than 20 years of experience was sought to judge.

### Statistical Analysis

We used descriptive analysis to summarize the demographic and clinical features of cases with various AEs resulting from the COVID-19 vaccine immunization in the VAERS database. We focused on three concerning AEs, including the anaphylactic response, concerning neurological disorders and death. Within the database, we compared the demographic and clinical characteristics between groups with and without such AEs using Fisher’s exact test. The statistical significance was determined at *P <0.017* with 95% confidence intervals since we conducted multiple comparisons. Data mining and the statistical analysis were performed by SPSS, version 21 (IBM Inc., NC, US). The figure was generated by GraphPad Prism, version 9.0 (GraphPad Software. LLC., CA, US).

## Results

### Demographic Analysis and Clinical Information

In December 2020, the VAERS had received 3.908 AE reports following COVID-19 mRNA vaccine immunization. We summarized the demographic features of the reports in **Table 1**. The average age for all AEFI cases was (42.75 ± 12.70) years. The majority of the affected cases were young (18–44 years old, 60.06%) and middle-aged (45–64 years old, 36.46%). Over 95% of the AE cases were reported from December 11th to December 30th, 2020, coinciding with the period when vaccines were being applied to the large-scale population. Within this period, most of the AEFIs (79.68%) were reported after the first dose of vaccination. A majority of cases (95.16%) received Pfizer-BioNTech vaccine, and 4.81% received Moderna vaccine. Almost 95% of the AEFIs occurred within three days after vaccination. We summarized the clinical characteristics in **Table S**, including recent illnesses, past medical histories, allergy histories, and currently administrated medications. Allergy histories were reported in more than half of the cases (50.29%) after removing unrecorded items. The allergy to antibiotics counted as the most common allergy category (26.48%).

**Table 1:** Demographic characteristics and vaccine information of cases with AEs after COVID-19 vaccine reported to VAERS database in December 2020.

Characteristics	Reports, no. (%)
Reporting states (top 5)	
California	370 (9.47)
Texas	288 (7.37)
Illinois	252 (6.45)
New York	165 (4.22)
Florida	154 (3.94)
Reporting date	
December 1–10, 2020	52 (1.33)
December 11–20, 2020	2,551 (65.28)
December 21–31, 2020	1,217 (31.14)
Unknown or missing	88 (2.25)
Sex of patients	
Male	756/3,844 (19.67)
Female	3,088/3,844 (80.33)
Unknown or missing	64/3,908 (1.64)
Age groups (years)	
<18	3/3,771 (0.08)
18–44	2,265/3,771 (60.06)
45–64	1,375/3,771 (36.46)
>65	128/3,771 (3.39)
Unknown or missing	137/3,908 (3.51)
Vaccine producer	
Pfizer	3,719 (95.16)
Moderna	188 (4.81)
Unknown or missing	1 (0.03)
Dosage	
First dose	3,114 (79.68)
Second dose	155 (3.97)
Unknown or missing	639 (16.35)
AE onset interval (days)	
0 day	2,686 (68.73)
1 day	838 (21.44)
2 days	134 (3.43)
3–7 days	52 (1.33)
8–14 days	2 (0.05)
15–21 days	9 (0.23)
Unknown or missing	131 (3.35)

COVID-19, coronavirus disease 19; AE, adverse event; VAERS, Vaccine Adverse Event Reporting System.

### AE Classifications by SOC Terms

**Figure 1:**
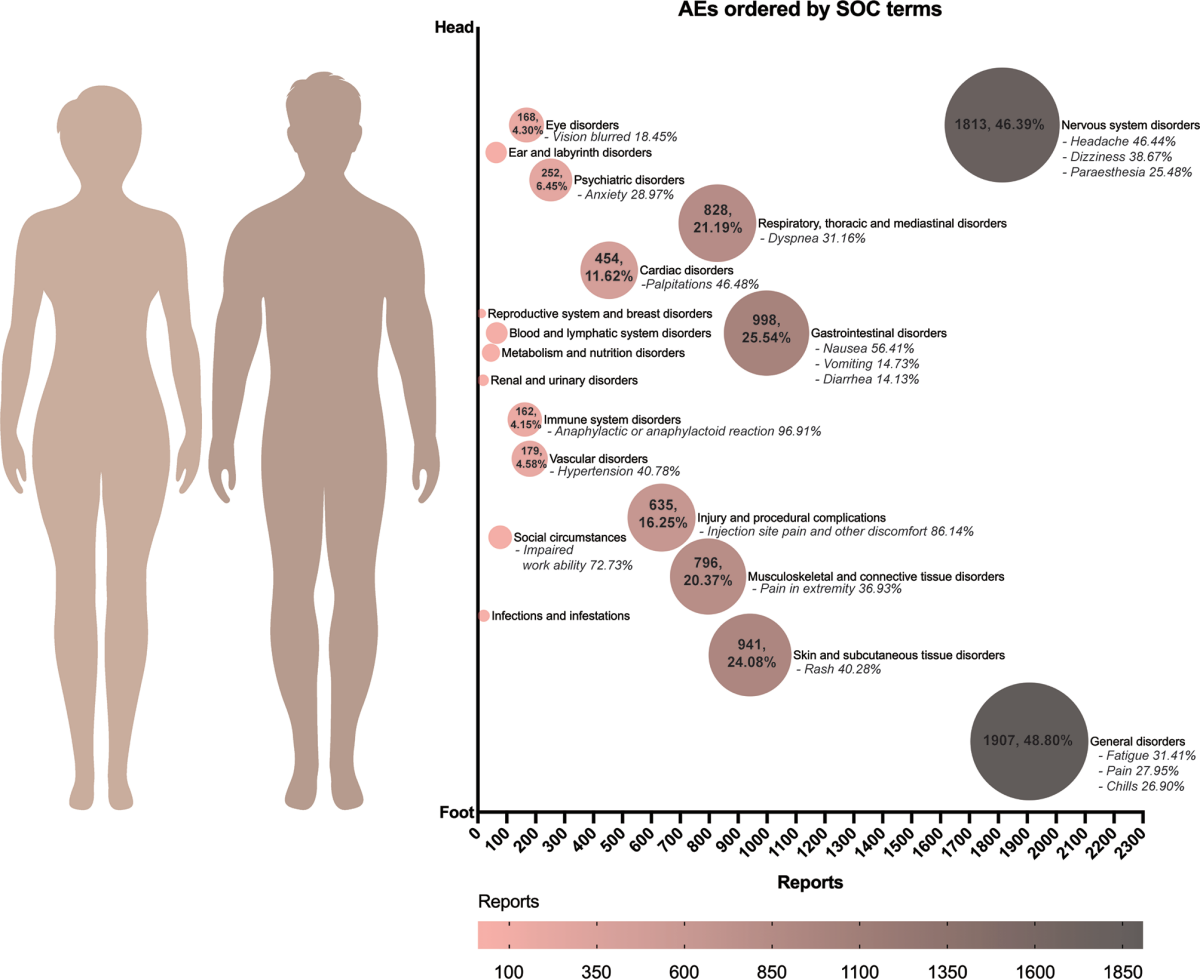
The AEs after COVID-19 mRNA vaccines classified with SOC terms in VAERS database in December 2020. The bubble plot showed the case numbers and percentages of different AEs among the total of people who reported AEs. AEs were generally sorted by organs from head to feet of a human body. More common AEs were represented with larger bubble sizes and darker colors. COVID-19, coronavirus disease 19; AE, adverse event; VAERS, Vaccine Adverse Event Reporting System.

We classified all AEFIs by SOC terms and generated **Figure 1**. Among the people who reported AEs, general disorders (48.80%), such as fatigue, pain, and chills, were the most common AEFIs. Nervous system disorders (46.39%) ranked as the second frequent AEFI. Among neurological disorders, symptoms like headache (46.39%), dizziness (38.67%), and paraesthesia (25.48%) were most commonly reported. However, we still identified some scarce but concerning nervous system disorders, such as syncope (2.04%), facial nerve paralysis (0.99%), and seizure (0.66%). We did not find Guillain-Barre syndrome in current VAERS reports. Gastrointestinal disorders (25.54%) followed as the third most common AEFI; nausea (56.41%), vomiting (14.73%), and diarrhea (14.13%) were most frequent in such AEs. We found 162 reports (4.15%) of immune system disorders, and 96.91% of them were suspected of anaphylactic or anaphylactoid reactions.

### Clinical Review of AEs Reports With Prespecified Conditions

To interpret the potentially associated conditions, we analyzed the recent illnesses, past histories, and allergy histories in some specified AEs, including immune system disorders, concerning neurological disorders (syncope, face paralysis, and seizure), and death (**Table 2**). The allergy history was generally more frequent in vaccine recipients with anaphylactic reactions than those without (64.91% *vs.* 49.62%, OR = 1.88, *P <0.017*). History of anxiety or depression was more common in subjects reporting severe neurological AEFIs, compared with those reporting other AEFIs (18.37% *vs.* 7.85%, OR = 2.64, *P <0.017*). Although we found no gender difference in cases with and without concerning neurological AEs, all 18 facial paralysis cases were notably females. We found 11 cases reporting a death in the VAERS database. The patients who died after vaccines were significantly older than other comparatives (79.36 ± 10.41 years old *vs.* 42.64 ± 12.55 years old, *P <0.01*, 95% CI 29.30–44.15). Meanwhile, they were more likely to experience hypertension (50.00% *vs.* 11.42%, OR = 7.76, *P <0.01*) and neurological disorders (50.00% *vs.* 5.36%, OR = 17.65, *P <0.01*), compared with other vaccine recipients.

**Table 2:** Comparison of characteristics in cases with AEs after COVID-19 vaccine sourced from the VAERS database in December 2020.

Characteristics	W/O immune disorders	Immune disorders	W/O concerning neurological symptoms	Concerning neurological symptoms	Alive	Dead
**Total N**	3,746	162	3,844	64	3,897	11
**Age (years, mean ± SD)**	42.73 ± 12.76	43.07 ± 11.30	42.80 ± 12.69	39.20 ± 12.97	42.64 ± 12.55	79.36 ± 10.41^**^
**Male, N/available N (%)**	736/3,685 (19.97)	20/159 (12.58)	745/3,781 (19.70)	11/63 (17.46)	751/3,833 (19.59)	5/11 (45.45)
**Recent diseases, N/available N (%)**	77/2,189 (3.52)	2/97 (2.06)	78/2,245 (3.47)	1/40 (2.44)	79/2,279 (3.47)	0/7 (0.00)
UTI	40/2,189 (1.83)	0/97 (0.00)	40/2,245 (1.78)	0/40 (0.00)	40/2,279 (1.76)	0/7 (0.00)
Other infections	27/2,189 (1.23)	1/97 (1.03)	28/2,245 (1.25)	0/40 (0.00)	28/2,279 (1.23)	0/7 (0.00)
**Past history, N/available N (%)**	1,519/2,631 (57.73)	69/118 (58.47)	1,565/2,700 (57.96)	23/49 (46.94)	1,580/2,741 (57.64)	8/8 (100.00)
Hypertension	307/2,631 (11.67)	10/118 (8.47)	311/2,700 (11.52)	6/49 (12.24)	313/2,741 (11.42)	4/8 (50.00)^**^
Diabetes	179/2,631 (6.80)	12/118 (10.17)	189/2,700 (7.00)	2/49 (4.08)	190/2,741 (6.93)	1/8 (12.50)
Neurological	142/2,631 (5.40)	9/118 (7.63)	148/2,700 (5.48)	3/49 (6.12)	147/2,741 (5.36)	4/8 (50.00)^**^
Ophthalmic	26/2,631 (0.99)	1/118 (0.85)	26/2,700 (0.96)	1/49 (2.04)	27/2,741 (0.99)	0/8 (0.00)
Thyroid	188/2,631 (7.15)	13/118 (11.02)	199/2,700 (7.37)	2/49 (4.08)	200/2,741 (7.30)	1/8 (12.50)
Asthma & COPD	320/2,631 (12.16)	16/118 (13.56)	335/2,700 (12.41)	1/49 (2.04)	335/2,741 (12.22)	1/8 (12.50)
CAD	124/2,631 (4.71)	5/118 (4.24)	127/2,700 (4.70)	2/49 (4.08)	127/2,741 (4.63)	2/8 (25.00)
Digestive	185/2,631 (7.03)	7/118 (5.93)	189/2,700 (7.00)	3/49 (6.12)	190/2,741 (6.93)	2/8 (25.00)
Kidney	30/2,631 (1.14)	2/118 (1.69)	31/2,700 (1.15)	1/49 (2.04)	31/2,741 (1.13)	1/8 (12.50)
CTD	98/2,631 (3.72)	6/118 (5.08)	103/2,700 (3.81)	1/49 (2.04)	104/2,741 (3.79)	0/8 (0.00)
Dermatological	54/2,631 (2.05)	5/118 (4.24)	57/2,700 (2.11)	2/49 (4.08)	59/2,741 (2.15)	0/8 (0.00)
Anemia	34/2,631 (1.29)	1/118 (0.85)	34/2,700 (1.26)	1/49 (2.04)	34/2,741 (1.24)	1/8 (12.50)
Coagulation	33/2,631 (1.25)	1/118 (0.85)	33/2,700 (1.22)	1/49 (2.04)	34/2,741 (1.24)	0/8 (0.00)
Psychiatric	214/2,631 (8.13)	7/118 (5.93)	212/2,700 (7.85)	9/49 (18.37)^*^	219/2,741 (7.99)	2/8 (25.00)
Tumor	58/2,631 (2.20)	2/118 (1.69)	59/2,700 (2.19)	1/49 (2.04)	60/2,741 (2.19)	0/8 (0.00)
**Allergy history, N/available N (%)**	1,244/2,507 (49.62)	74/114 (64.91)^*^	1,297/2,574 (50.39)	21/47 (44.68)	1,316/2,613 (50.36)	2/8 (25.00)
Antibiotics	660/2,507 (26.33)	34/114 (29.82)	681/2,574 (26.46)	13/47 (27.66)	693/2,613 (26.52)	1/8 (12.50)
Other vaccines	34/2,507 (1.36)	2/114 (1.75)	36/2,574 (1.40)	0/47 (0.00)	36/2,613 (1.38)	0/8 (0.00)
Contrast	42/2,507 (1.68)	4/114 (3.51)	46/2,574 (1.79)	0/47 (0.00)	46/2,613 (1.76)	0/8 (0.00)
Fruits	88/2,507 (3.51)	3/114 (2.63)	90/2,574 (3.50)	1/47 (2.13)	91/2,613 (3.48)	0/8 (0.00)
Seafood	111/2,507 (4.43)	6/114 (5.26)	113/2,574 (4.39)	4/47 (8.51)	117/2,613 (4.48)	0/8 (0.00)
Pets and insects	96/2,507 (3.83)	7/114 (6.14)	102/2,574 (3.96)	1/47 (2.13)	103/2,613 (3.94)	0/8 (0.00)
Plants and pollen	91/2,507 (3.63)	4/114 (3.51)	95/2,574 (3.69)	0/47 (0.00)	95/2,613 (3.64)	0/8 (0.00)
Metal	19/2,507 (0.76)	1/114 (0.88)	19/2,574 (0.74)	1/47 (2.13)	20/2,613 (0.77)	0/8 (0.00)
Other objects	211/2,507 (8.42)	11/114 (9.65)	221/2,574 (8.59)	1/47 (2.13)	222/2,613 (9.22)	0/8 (0.00)

*<0.017, **<0.01, values in column immune disorders compared with values in column W/O immune disorders; values in column concerning neurological symptoms compared with values in column W/O neurological symptoms; values in column dead compared with values in column alvie. COVID-19, coronavirus disease 19; AE, adverse event; VAERS, Vaccine Adverse Event Reporting System; W/O, without; N, number; UTI, upper respiratory tract infection; COPD, chronic obstructive pulmonary disease.

### Prognosis Due to AEs Following COVID-19 Vaccines

To analyze the prognosis of AEs following the COVID-19 vaccines, we assessed the recovery rates and visit rates in various medical facilities in the VAERS database of December 2020 (**Table 3**). Except for the unreported cases, 65.12% of vaccine recipients reported recovery from AEs. Generally, the outpatient and emergency room visit rates were 11.92 and 22.42% for all AEFIs. Only 2.53% of all AEFI cases within the current VAERS database required hospitalization. More than one-third of the hospitalized patients were recovered. A tiny proportion of the reports belonged to life-threatening AEs (0.97%) or ended up with death (0.28%) among all AE cases. For the 38 cases that developed life-threatening AEs, around 50% of them have reported recovery. To be noted, the 11 death cases reported in the VAERS database could not set up the causal relationship with the vaccine by interpreting the descriptions in the database. More details were still needed to analyze the cause of death.

**Table 3:** Outcomes of patients with AEs after COVID-19 vaccine sourced from the VAERS database in December 2020 (updated to December 30, 2020).

Outcomes	Total	Outpatient	ER visit	Hospitalized	Life-threatening	Death
**Total N**	3,908	466	876	99	38	11
**Recovered,** **N (%)**	1,785 (45.68)	184 (39.48)	481 (54.91)	35 (35.35)	17 (44.74)	NA
**Not recovered,** **N (%)**	956 (24.46)	150 (32.19)	124 (14.16)	38 (38.38)	14 (36.84)	11(100.00)
**Unknown,** **N (%)**	1,167 (29.86)	132 (28.33)	271 (30.94)	26 (26.26)	7 (18.42)	NA

COVID-19, coronavirus disease 19; AE, adverse event; VAERS, Vaccine Adverse Event Reporting System; ER, emergency room; N, number.

## Discussion

Our study focused on the AEFIs and completed a comprehensive collection until recently to demonstrate the severity of AEs and possible connection with basic clinical features. We confirmed the favorable safety profiles of mRNA vaccines observed in a series of clinical trials (4, 5, 12).

According to the SOC terms, we organized all AEs reported to VAERS to narrow the heterogeneity in their original descriptions. Our classification signaled that general disorders, dominated by fatigue, pain, and chills, took over almost half of the AEFIs among the reported AE cases. Nervous system disorders represented by headache and dizziness, and some other reactions like nausea, vomiting, rash, pain in extremity, and injection site pain, were also frequent within the people who reported AEs. These findings reassuringly echoed previous clinical studies results, which demonstrated that local reactions were common; Simultaneously, systemic side effects, such as fatigue, myalgia, arthralgia, and headache, were noted in about 50% of participants (4, 5).

Severe allergic reaction, including anaphylaxis, is a crucial issue related to a newly licensed vaccine. Anaphylaxis could be life-threatening, typically onsets within minutes to hours after vaccination (13). We found 157 VAERS reports of possible anaphylactic or anaphylactoid reactions during our analytic period; however, the definitively confirmed diagnoses could be fewer. Unlike some common symptoms such as fatigue and headache, the diagnosis of anaphylaxis requires professional judgment, such as case-by-case interpretation with Brighton Collaboration case definition criteria (14). In an early safety monitoring of the BNT162b2 vaccine, 21 cases were determined to be true anaphylaxis among 175 anaphylactoid reaction reports screened in the VAERS database; the anaphylaxis rate was as low as 11.1 cases per million vaccine dose administered (7). Similar to the previous studies (6, 7), we found COVID-19 vaccine recipients with immune response were more likely with a history of allergies. It is vital to screen contraindications, prepare necessary supplies in the vaccination site, and implement adequate postvaccination observation to prevent severe allergies.

Previous studies noted some rarer neurological findings, such as facial paralysis (4, 5, 9, 10). The BNT162b2 and mRNA-1273 phase 3 trials reported seven cases with suspected facial paralysis in 36,930 vaccine receivers (incidence rate 0.019%) (4, 5). However, in the scenario of rare and potential AE issues, clinical trials lack enough power to draw a definitive conclusion due to their strict inclusion criteria, limited sample sizes, and relatively short observation periods. The SRS could be a supplementary source for new evidence. Taking advantage of VAERS, we identified 18 cases with facial paralysis, together with other concerning neurological events like syncope (37 cases) and seizure (12 cases). Such concerning neurological AEs after vaccines may be more than chance events, and the possibility and causality bear close monitoring. A VAERS analysis previously reported the possible relationship between facial paralysis (0.05% incidence rate) and influenza vaccine (15). The female prevalence in all-cause facial paralysis was 58.2–65.8% in some previous studies (16–18); however, the 18 AEFI cases with facial paralysis were notably all females in our study. We further analyzed that neurological AEs were more likely to happen in vaccine recipients with emotional distress histories. Some neurological diseases and emotional disorders share mutual risk factors. A case-control study hinted that psychological stress could exacerbate facial paralysis (19), and another retrospective cohort demonstrated a bidirectional association between facial paralysis and anxiety disorders (20). As a public health emergency, the COVID-19 outbreak psychologically impacted a massive population, resulting in moderate-to-severe anxiety in about one-third of respondents in an online survey (21). If there were possible links between vaccination and some neurological AEs, psychological support should be emphasized, especially for patients with histories of emotional disorders.

Within the current VAERS database, the hospitalization rate (2.53%) and cases that resulted in life-threatening AEs (0.97%) were minor among all 3,908 COVID-19 mRNA vaccine recipients. We could not calculate the accurate recovery rate of AEFI cases due to the un-reporting records in the database, and the results labeled unrecovered were possibly not final due to the relatively short analytic period. Overall, the prognosis of AEFIs after the COVID-19 vaccines could be favorable based on some previous studies demonstrating the complete recovery for all anaphylaxis cases (6, 7). The BNT162b2 and mRNA-1273 phase 3 trials separately noted two participants died in vaccine arms without clear causality with vaccines (4, 5). We identified 11 cases (0.28%) that reported death in the VAERS database. Still, we could not set up causality after reviewing the description case by case. However, we found that those who died after the vaccine were significantly older (79.36 ± 10.41-year-old *vs.* 42.64 ± 12.55-year-old, *P <0.01*) and had more underlying diseases compared to other vaccine recipients. Since previous clinical trials included limited old participants with relatively more risk factors (4, 5), a more desired vaccine dosing and the procedure is needed for the elderly.

It was demonstrated that systemic reactogenicity was more common and severe after receiving the second dose than the first (4). However, most AEs in our observations occurred after receiving the first dose since most vaccine recipients completed only the first dose of vaccine during our analytic period. A convincing real-world comparison of reaction of the first and second vaccine doses could be available when the reported cases grow in the future VAERS database.

We admit some limitations to our study. First, unlike researchers in clinical trials who report AEs using standardized data collection methods, the quality of information submitted by VAERS reporters varies widely. The organization of SOC terms merits, but it does not work for some vague descriptions. Second, as a passive SRS, VAERS is inherited with reporting biases, over-reporting or under-reporting, and lack of an unvaccinated comparison group (11). Although it is less likely to under-report a concerning AE, some mild or moderate AEs may actually be more frequent than what we assessed in the database. Third, VAERS does not provide denominator data to calculate the incidence rates of AEs (22); therefore, we could not calculate accurate incidence rates by interpreting the VAERS database. Furthermore, the quality and completeness of the reports collected in VAERS are variable, and some records lack valid medical diagnoses, thus making the assessment of causality challenging. Although we made an effort to demonstrate the possible connection between certain AEFIs and clinical backgrounds, the causal relationship could not be confirmed.

## Conclusion

We confirmed the AEFIs associated with the COVID-19 mRNA vaccines were generally mild treatable local or systemic reactions based on the VAERS real-world data. We suggest that the vaccine recipients with past allergy histories should be observed closely to prevent severe allergies, and patients with a prior history of anxiety need to be evaluated before vaccination and monitored for neurological symptoms after vaccination. Moreover, elderly persons with multiple underlying diseases need a repeated evaluation and a more desired vaccination procedure. Our findings pave the way for continued AEFI investigation. We recommend assessing the detailed profiles of vaccine recipients who developed severe AEFIs to explore the potential causal relationship between clinical characteristics and symptoms after the vaccine.

## Data Availability Statement

The original contributions presented in the study are included in the article/**Supplementary Material**. Further inquiries can be directed to the corresponding author.

## Ethics Statement

Ethical review and approval were not required for the study on human participants in accordance with the local legislation and institutional requirements. Written informed consent for participation was not required for this study in accordance with the national legislation and the institutional requirements.

## Author Contributions

BZ and GC designed the study. GC, XiL, MY, and MS analyzed and interpreted data. GC generated figures/tables and drafted the manuscript. YZ and XuL reviewed and corrected the manuscript. All authors contributed to the article and approved the submitted version.

## Funding

This work has been made possible through an ISN Sister Renal Centers Grant.

## Conflict of Interest

The authors declare that the research was conducted in the absence of any commercial or financial relationships that could be construed as a potential conflict of interest.

## Abbreviations

COVID-19, coronavirus disease 19; AE, adverse event; AEFI, adverse event following immunization; RCT, randomized controlled trial; VAERS, Vaccine Adverse Event Reporting System; FDA, Food and Drug Administration; SRS, spontaneous reporting system; CDC, Centers for Disease Control and Prevention; MedDRA, Medical Dictionary for Regulatory Activities; SOC, system organ classification.
