# Supplementary material for: COVID-19 mRNA Vaccines Are Generally Safe in the Short Term: A Vaccine Vigilance Real-World Study Says
Source: Front Immunol. 2021 May 21;12:669010. doi: 10.3389/fimmu.2021.669010 (PMC8177815; doi:10.3389/fimmu.2021.669010)
Supplement: Supplementary file 1 [file Table_1.docx]

**Table S:** Clinical characteristics of cases with AEs after the Covid-19 vaccine reported to VAERS database in December 2020.

| **Characteristics** | **Reports, no. (%)** |
| --- | --- |
| **Recent diseases** | 79/2286 (3.46) |
| Upper respiratory tract infection | 40 (50.63) |
| Other non-severe infection | 28 (35.44) |
| Other diseases | 11 (13.92) |
| **Past history** | 1588/2749 (57.76) |
| Hypertension | 317 (11.53) |
| Diabetes | 191 (6.95) |
| Other metabolic diseases | 140 (5.09) |
| Neurological diseases | 151 (5.49) |
| Ophthalmic diseases | 27 (0.98) |
| Thyroid diseases | 201 (7.31) |
| Asthma and chronic obstructive pulmonary disease | 336 (12.22) |
| Cardiac diseases | 129 (4.69) |
| Digestive diseases | 192 (6.98) |
| Kidney diseases | 32 (1.16) |
| Connective tissue diseases | 104 (3.78) |
| Dermatological diseases | 59 (2.15) |
| Anemia | 35 (1.27) |
| Coagulation disorders | 34 (1.24) |
| Psychiatric disorders | 221 (8.04) |
| Tumor | 60 (2.18) |
| Others | 110 (4.00) |
| **Allergy history** | 1318/2621 (50.29) |
| Allergy to antibiotics | 694 (26.48) |
| Allergy to other vaccines | 36 (1.37) |
| Allergy to contrast | 46 (1.76) |
| Allergy to other medications | 470 (17.93) |
| Allergy to fruits | 91 (3.47) |
| Allergy to seafood | 117 (4.46) |
| Allergy to other food | 201 (7.67) |
| Allergy to pets and insects | 103 (3.93) |
| Allergy to plants and pollen | 95 (3.62) |
| Allergy to metal | 20 (0.76) |
| Allergy to other objects or environment | 222 (8.47) |
| **Current medications** | 1754 (44.88) |
| Diet supplements and vitamins | 689 (17.63) |
| Sedative-hypnotic medications | 539 (13.79) |
| Antihypertensives | 402 (10.29) |
| Endocrine system medications | 379 (9.70) |
| Antiallergic medications | 377 (9.65) |
| Analgesics | 300 (7.68) |
| Digestive system medications | 262 (6.70) |
| Cardiac system medications | 255 (6.53) |
| Bronchodilators | 224 (5.73) |
| Contraceptive medications | 224 (5.73) |
| Nervous system medications | 118 (3.02) |
| Antimicrobial agents | 86 (2.20) |
| Immunosuppresive agents | 71 (1.82) |
| Dermatologic agents | 32 (0.82) |
| Ophthalmic medications | 11 (0.28) |
| Antitumor medications | 7 (0.18) |
| Others | 175 (4.48) |

Abbreviations: Covid-19: coronavirus disease 19; AE: adverse event; VARES: Vaccine Adverse Event Reporting System
